# Discordance between ^90^Y-PET/CT(MR)-estimated activity and dose calibrator measured administered activity: an international study in SIRT patients treated with resin and glass microspheres

**DOI:** 10.1186/s40658-025-00725-8

**Published:** 2025-02-05

**Authors:** Thomas Carlier, Silvano Gnesin, Justin K. Mikell, Maurizio Conti, John O. Prior, Niklaus Schaefer, Maria del Sol Pérez Lago, Clément Bailly, Yuni K. Dewaraja, Thiago V. M. Lima

**Affiliations:** 1 Nuclear Medicine Department, University Hospital of Nantes, Nantes, France; 2 Institute of Radiation Physics, Lausanne University Hospital and University of Lausanne, Lausanne, Switzerland; 3 Department of Radiation Oncology, Washington University in St. Louis, St. Louis, MO USA; 4 Siemens Medical Solutions, Knoxville, TN USA; 5 Department of Nuclear Medicine and Molecular Imaging, Lausanne University Hospital and University of Lausanne, Rue du Bugnon 46, 1011 Lausanne, Switzerland; 6 Department of Radiology and Nuclear Medicine, Luzerner Kantonsspital, Lucerne, Switzerland; 7 Division of Nuclear Medicine, Department of Radiology, University of Michigan, Ann Arbor, MI USA

**Keywords:** Resin microspheres, Glass microspheres, Yttrium-90, PET/CT, Activity

## Abstract

**Purpose:**

Therapeutic administration of ^90^Y-loaded microspheres is routinely used for primary and secondary liver tumours. For activity-based therapeutic prescription the activity must be within 10% of the intended activity. Previous studies reported significant discrepancies between manufacturer-declared vial activities and both experimental and Monte-Carlo assessments, greater than 10%, for resin/glass ^90^Y-microspheres. The objective of this work was to investigate whether these discrepancies were also seen in patients.

**Methods:**

We analysed patient ^90^Y-PET reconstructions (99 glass and 15 resin microspheres) from 4 different institutions and 4 different systems. We considered tail-fitting background scaling (TFBS) and absolute scaling (ABS), for scatter correction. Residuals after therapeutic injection were measured. Eighty-one patients were imaged with PET/CT and 33 with PET/MR. The PET measured activity (A_PET_) was assessed in the whole liver. The ratio A_PET_/A_calibrator_ was calculated for each patient, where A_calibrator_ was the injected activity measured by the dose calibrator corrected for residual and lung shunt.

**Results:**

Quantification ratio between calibrators and PET was significantly different from 1, regardless of the scatter correction used. In glass microspheres, the mean A_PET/CT_/A_calibrator_ was 0.84 ± 0.06 for TFBS and 0.90 ± 0.06 for ABS (0.66 ± 0.09 and 0.76 ± 0.07 for (A_PET/MR_/A_calibrator_)). The mean A_PET/CT_/A_calibrator_ ratio for resin microspheres was 1.16 ± 0.09 for TFBS and 1.30 ± 0.12 for ABS.

**Conclusions:**

We observed in patients similar activity discrepancies as reported for vials, with a relative difference of 44 ± 16% between glass and resin ^90^Y-loaded microspheres. In ^90^Y hepatic radioembolization, the 10% accuracy prerequisite on knowing the administered therapeutic activity is then unlikely to be met.

**Supplementary Information:**

The online version contains supplementary material available at 10.1186/s40658-025-00725-8.

## Introduction

Permanent implantation of ^90^Y-loaded microspheres is routinely used in selective internal radiotherapy of primary and secondary liver tumours. In this context, the estimation of absorbed dose (AD) to tumour and healthy liver has been shown to be clinically effective for both ^90^Y-loaded glass (TheraSphere®, Boston Scientific) [1] and ^90^Y-loaded resin (SIR-Spheres®, Sirtex Medical) microspheres [2] in patients treated with Selective Internal Radiation Therapy (SIRT) for locally advanced hepatocellular carcinoma (HCC). Therefore, current international recommendation on SIRT procedures consider dosimetry as mandatory or strongly recommended [3–5].

The activity present in the imaged perfused volumes is derived from the vendor calibration. Consequently, any quantitative bias affecting the accuracy of the manufacturer-calibrated activity leads to a potential bias in the predictive AD assessments. Furthermore, there have been increasing efforts to use PET imaging to estimate the actual post-treatment AD and derive the effective correlation with the patient outcome [6–8].

Three recent studies have reported that the vial activity declared by the manufacturer was significantly overestimated/underestimated for glass and resin microspheres, respectively, by comparing the vial activity reported on the calibration certificate provided by the manufacturer and the vial activity measured in a high-purity germanium well-counter [9] or using ^90^Y-PET imaging [10]. Experimental PET findings were then corroborated by Monte-Carlo simulations [11]. Such discrepancy has also been observed in patients using quantitative ^90^Y-SPECT/CT imaging in SIRT with resin and glass microspheres [12], although quantitative measurement with ^90^Y-SPECT/CT can be challenging due to the complexities of imaging bremsstrahlung photons.

The reported discrepancies between vendor stated activities and independent evaluations, motivated further investigations of the concordance between the claimed vendor calibrated ^90^Y activity and an independent assessment in a clinically relevant setting, i.e. post-administration patient ^90^Y-PET/CT imaging, across multiple centres and imaging systems. In this study, we compared the ^90^Y activity in the liver of 114 patients (treated with resin or glass ^90^Y-loaded microspheres) assessed with PET in 4 institutions and 6 scanners with the intended administered activity measured in dose calibrators traceable to the vendors standard. We also evaluated how the discrepancies between dose calibrator (i.e. the vendor reference) and quantitative PET were affected by using two different background scaling methods for scatter estimation in the single scatter simulation (SSS) method because this can lead to substantial inaccuracies in the reconstructed images under low count-conditions encountered in ^90^Y-PET/CT [13, 14].

## Material and methods

Patients (number treated with glass microspheres: 99, resin microspheres: 15) were retrospectively enrolled from the 4 different institutions with various clinical indications, including primary and secondary liver tumours. Only patients with a lung shunt fraction (LSF) of less or equal than 5% (measured by either planar or SPECT/CT imaging during the work-up) were included, to avoid significant impact of this measurement on our results as LSF measurement is associated with high uncertainty. The clinical indications are reported in Table 1 while the total number of patients for each institution as a function of microspheres’ type and imaging system used is shown in Table 2. In the University Hospital of Nantes ethical approval was waived by the local Ethics Committee (*Groupe Nantais d’Éthique dans le Domaine de la Santé*) in view of the retrospective nature of the study and all procedures being performed were part of the routine care. All the patients from CHUV participating in this retrospective study have signed or did not oppose to the general consent form for research and for retrospective use of their images for clinical research. A protocol was submitted to CER-VD (protocol 2018-01513). All LUKS’ patients presented in this study have signed a general consent for retrospective use of their images for clinical research. Finally, all patients at University of Michigan signed an informed consent for ^90^Y-PET/CT imaging approved by the Institutional Review Board (protocols HUM00118705 and HUM00181352).

**Table 1:** Population characteristics

Characteristics	Study population (n = 114)		
Age (y)*	70 [49–88]		
Women (%)	27		
BMI (kg/m^2^)*	26.1 [17.5–38.7]		
Type of microspheres		*Glass*	*Resin*
Hepatocellular carcinoma**	78	70 (90%)	8 (10%)
Cholangiocarcinoma**	10	9 (90%)	1 (10%)
Liver metastases (colorectal cancer)**	5	1 (20%)	4 (80%)
Liver metastases (neuroendocrine)**	16	16 (100%)	0 (0%)
Liver metastases (other)**	8	6 (75%)	2 (25%)

*Expressed as median, with range in square brackets

**Number of patients

**Table 2:** Total number of patients per institution

Institution	Microspheres type	PET scanner	Number of patients
CHUV	Glass	Vision 600 (PET/CT-1)	16
	Resin	Vision 600 (PET/CT-1)	2
University of Michigan	Glass	Biograph mCT (PET/CT-2)	23
Luzerner Kantonsspital	Resin	Vision 600 (PET/CT-3)	5
University Hospital of Nantes	Glass	Vision 600 (PET/CT-4)	30
		Biograph mMR (PET/MR-1)	30
	Resin	Vision 600 (PET/CT-4)	5
		Biograph mMR (PET/MR-1)	3

### Administered activity measurement with dose calibrator

The model of dose calibrator used by each centre was: Veenstra VDC-606 (CHUV), CAPINTEC 25R (University of Michigan), Veenstra VDC-606 (Luzerner Kantonsspital) and Scintidose, Lemer Pax (University Hospital of Nantes). A local calibration factor was first set for each microsphere type based on the manufacturer’s recommendation and a calibrated source. Quality control was performed every day to test constancy of the dose calibrator settings. For all patients included in the analysis, the local versus manufacturer certified activity was found to be within 5% at all centres when comparing the manufacturer calibrated activity and the locally measured activity.

### Quantitative PET and comparison with calibrator measured activity

Of note, all PET scanners used in the current study allowed for native ^90^Y quantification, considering the physical decay of ^90^Y (64.2 h) and the positron branching ratio (32 × 10^–6^). ^90^Y-PET images were acquired and reconstructed in activity units (Bq/mL) using the 4 different systems described above according to local procedures (Table 3). For each patient, two different methods of SSS background scaling were considered: tail-fitting background scaling (TFBS) and absolute scaling (ABS) [15] which is thought to be more appropriate for low-count data such as those found in ^90^Y-PET. Residual activity (A_residual_) after therapeutic injection was measured either with a survey-meter (University of Michigan; N = 23) following vendor instructions or by PET/CT imaging (CHUV, Luzerner Kantonsspital and University Hospital of Nantes; N = 91) (Supplemental Table 1). For the survey meter approach, all contaminated waste (vial, tubing, catheter, etc.) was placed in the NaLgene container with an average exposure rate reading for 4 cardinal angles. The estimate of the residual activity was then calculated by comparing the average exposure rate from the waste to the decay corrected exposure rate of the initial vial at 30 cm.

**Table 3:** Acquisition and reconstruction parameters for each system

PET ID	Device	Centre	Acquisition parameters	Reconstruction parameters
PET/CT-1	Siemens Biograph Vison 600	CHUV Lausanne	List-mode 15 min (step and shoot)	OP-OSEM3D TOF + PSF 2 iterations × 5 subsets; Gaussian post-filtering (FWHM = 4 mm) Voxel size = 3.3 × 3.3x1.65 mm^3^
PET/CT-2	Siemens Biograph mCT 40	University of Michigan Hospital	List-mode 20 min (step and shoot)	OP-OSEM3D TOF + PSF 2 iterations × 21 subsets; Gaussian post-filtering (FWHM = 5 mm) Voxel size = 4 × 4 × 2 mm^3^
PET/CT-3	Siemens Biograph Vison 600	Luzerner Kantonsspital	List-mode 15 min (step and shoot)	OP-OSEM3D TOF + PSF 2 iterations × 5 subsets; Gaussian post-filtering (FWHM = 4 mm) Voxel size = 3.3 × 3.3x1.65 mm^3^
PET/CT-4	Siemens Biograph Vison 600	University Hospital of Nantes	List-mode 30 min (step and shoot)	OP-OSEM3D TOF + PSF 2 iterations × 5 subsets; Gaussian post-filtering (FWHM = 4 mm) Voxel size = 3.3 × 3.3 × 1.65 mm^3^
PET/MR-1	Siemens Biograph mMR	University Hospital of Nantes	List-mode 30 min (step and shoot)	OP-OSEM3D PSF 3 iterations × 21 subsets; Gaussian post-filtering (FWHM = 4 mm) Voxel size = 4.2 × 4.2 × 2 mm^3^

The PET measured activity (A_PET_) was assessed in a volume encompassing the liver (CT/MR-based segmentation) expanded by 1 cm to account for resolution and motion spill-out effects. The A_PET_/A_calibrator_ ratio was then calculated for each patient, where A_calibrator_ was the injected activity as measured by the local dose calibrator, corrected for residuals and LSF. The dial setting of the local dose calibrator was first set for each microspheres’ type based on the manufacturer’s calibrated activity. The residual fraction was defined as A_residual_/A_PET_.

### Statistical analysis

Wilcoxon signed-ranked test was used for all comparisons. A significant difference was considered when p < 0.05. A Bland–Altman method was used to analyse the difference between TFBS and ABS.

## Results

Table 4 presents the summary statistics for the injected activity, LSF and residual fraction as a function of the type of microspheres used.

**Table 4:** Summary statistics for the entire population

	Microspheres’ type	Mean	Range
Activity @ PET imaging time (MBq)	Glass	2775	358–8740
	Resin	1221	720–1907
LSF (%)	Glass	1.6	0.1–5
	Resin	1.4	0–3.8
Residual fraction (%)	Glass	2.2	0–21.8
	Resin	5.8	1.4–11.2
Liver volume (mL)		1932	552–5960

LSF: Lung shunt fraction

**Fig. 1:**
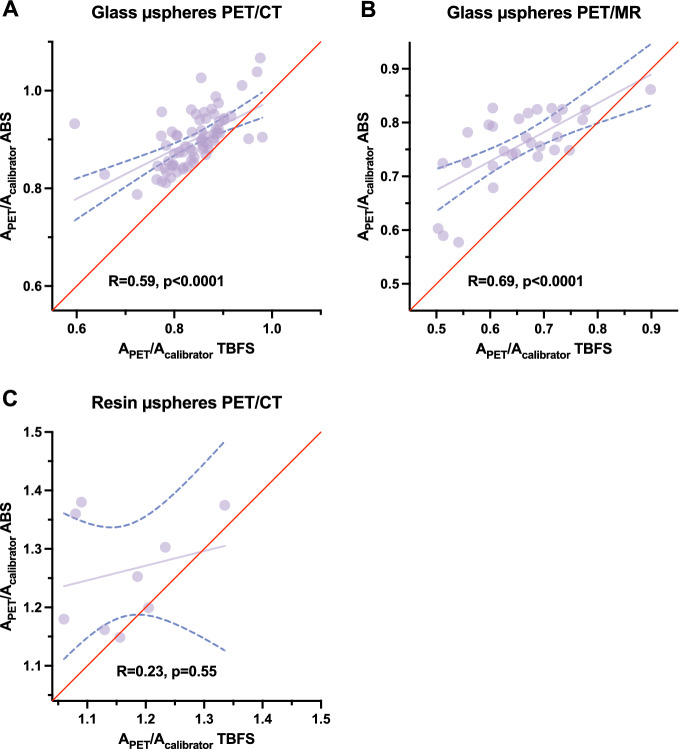
Correlation between A_PET_/A_calibrator_ computed using TFBS and ABS for ^90^Y-loaded glass microspheres using PET/CT (**A**) and PET/MR (**B**); and ^90^Y-loaded resin microspheres using PET/CT (**C**). The red line is the line of identity

Figure 1 shows the correlation between A_PET_/A_calibrator_ calculation using TFBS and ABS for PET/CT and PET/MR. For PET/CT systems, the mean A_PET_/A_calibrator_ was 0.84 (range: 0.60–0.98) when using TFBS and 0.90 (range: 0.79–1.07) when using ABS for ^90^Y-loaded glass microspheres and this difference was significant (Supplemental Figure S1, p < 0.0001). The same conclusions were drawn for the PET/MR system: the mean A_PET_/A_calibrator_ ratio was 0.66 (range: 0.50–0.90) when using TFBS and 0.76 (range: 0.58–0.86) when using ABS for glass microspheres and this difference was significant (Supplemental Figure S1, p < 0.0001). The same trend was observed for ^90^Y-loaded resin microspheres, although the number of patients was limited, with a mean A_PET_/A_calibrator_ of 1.16 (range: 1.06–1.34) when using TFBS and 1.30 (range: 1.15–1.50) when using ABS and this difference was significant (p < 0.01). The Bland–Altman plots for scatter fraction using ^90^Y-loaded glass microspheres and PET/CT systems are shown in Supplemental Figure S2.

**Fig. 2:**
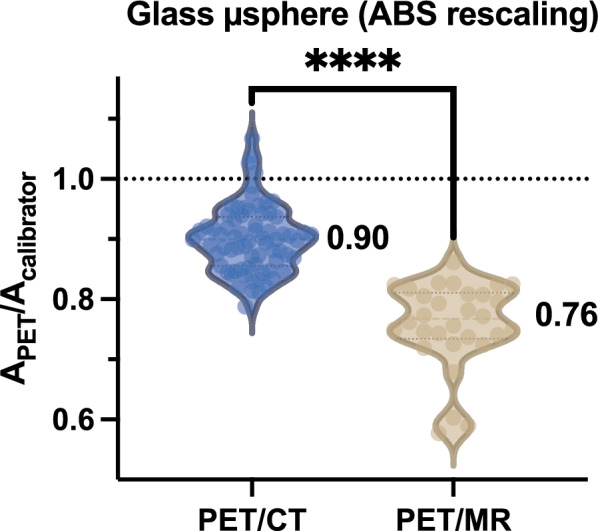
Comparison of A_PET_/A_calibrator_ between patients acquired using a PET/CT and those with the PET/MR (patients are different between PET/MR and PET/CT, except one). Only patients treated with ^90^Y-loaded glass microspheres are considered and data were reconstructed using the absolute rescaling for scatter estimation

Although patients imaged using PET/CT systems and PET/MR were not the same (except for one), a comparison of A_PET_/A_calibrator_ between the two modalities for ^90^Y-loaded glass microspheres (Fig. 2) showed a significant difference between the two modalities (p < 0.0001).

**Fig. 3:**
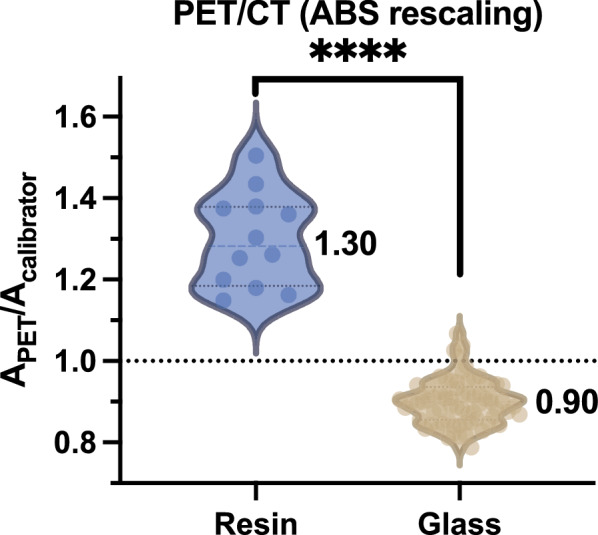
Comparison of A_PET_/A_calibrator_ between patients treated with ^90^Y-loaded glass and resin microspheres imaged using a PET/CT (absolute rescaling for scatter estimation). Results with TFBS scatter estimation are in Supplemental Figure S6

The comparison between A_PET_/A_calibrator_ for ^90^Y-loaded glass and resin microspheres is displayed in Fig. 3 when using PET/CT systems (reconstruction performed with ABS for scatter estimation). The A_PET_/A_calibrator_ was 0.90 ± 0.06 and 1.30 ± 0.12 for, respectively, ^90^Y-loaded glass and ^90^Y-loaded resin microspheres with significant statistical difference (p < 0.0001).

**Fig. 4:**
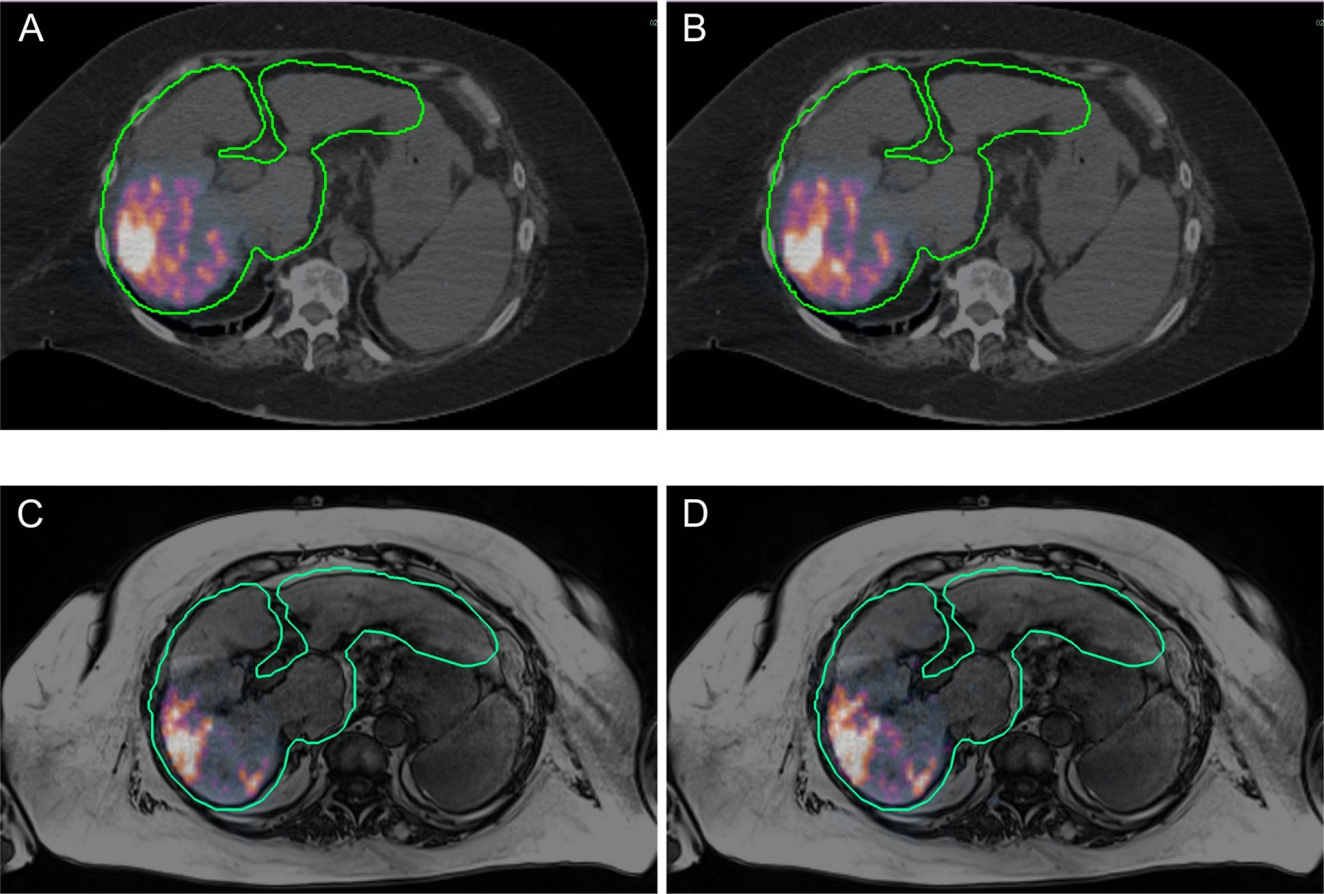
Patient with Child Pugh B7 cirrhosis treated for an HCC of the right posterior sector of the liver with ^90^Y-loaded glass microspheres (BMI = 24.7 kg m^−2^) and imaged with PET/CT (**A**, **B**) and PET/MR (**C**, **D**). Images were acquired on the same day for both modalities. The injected activity was 2001 MBq. The reconstructed activity in the liver with the 1-cm expanded contour was 1888 MBq (difference of − 5% compared with injected activity) using TFBS and PET/CT (**A**), 1788 MBq (− 10%) using ABS and PET/CT (B), 1103 MBq (− 44%) using TFBS and PET/MR and 1439 MBq (–27%) using ABS and PET/MR. The computed scatter fractions were 43%, 46%, 72% and 45% for (**A**), (**B**), (**C**) and (**D**), respectively. The PET image contrast is identical between all images

**Fig. 5:**
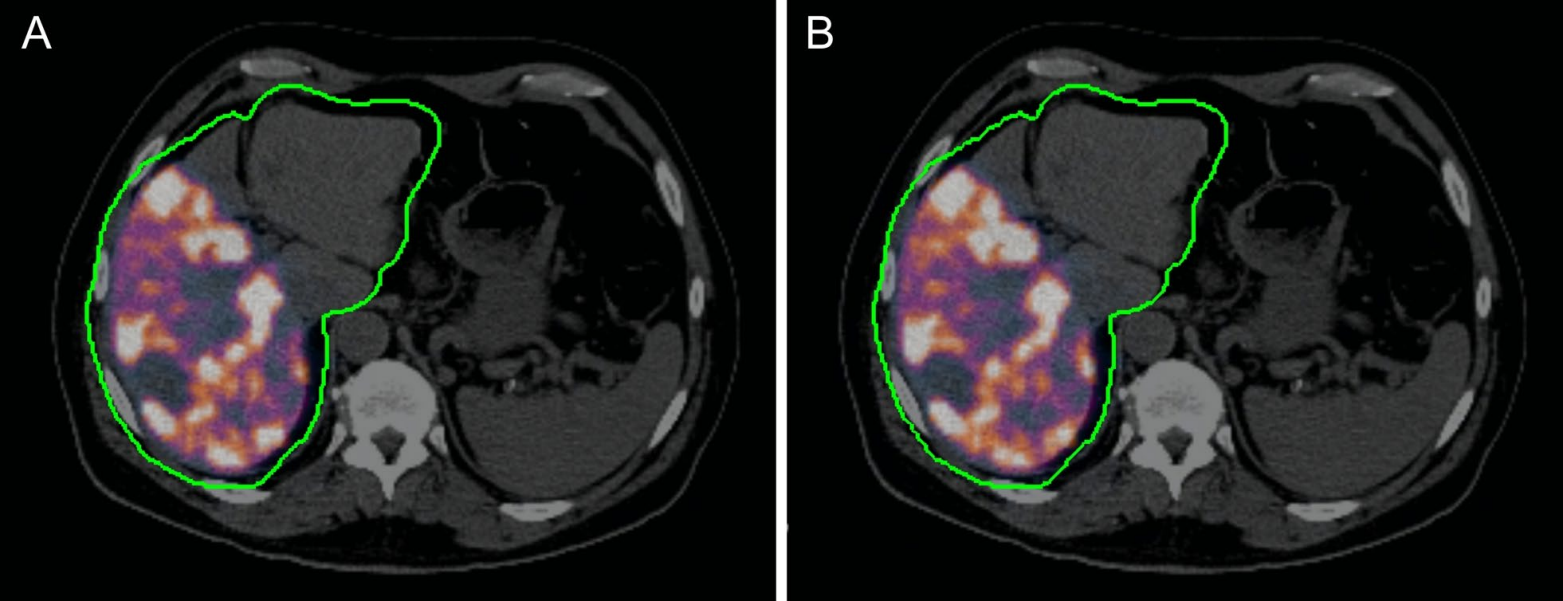
Patient treated for an HCC of the segment V of the liver with ^90^Y-loaded resin microspheres (BMI = 28.7 kg m^−2^) injected in the right paramedian sectoral arterial branch. The injected activity was 1170 MBq. The reconstructed activity in the liver (residual fraction of 11% accounted for) was 1361 MBq (+ 19%) using TFBS and PET/CT (**A**), 1437 MBq (+ 25%) using ABS and PET/CT (**B**). The computed scatter fractions were 57% and 41% for (**A**) and (**B**), respectively. The PET image contrast is identical between all images

Figure 4 (respectively, Fig. 5) shows an example of a patient treated with ^90^Y-loaded glass microspheres (respectively, ^90^Y-loaded resin microspheres)

## Discussion

Recent studies have shown significant discrepancies between the ^90^Y microspheres activity reported by the manufacturer and the activity measured in-situ prior to injection into the patient for both ^90^Y-loaded glass and resin microspheres [9–11]. In a separate work, we reported an average A_PET_/A_calibrator_ of 0.79 ± 0.04 for ^90^Y-loaded glass microspheres (i.e. overestimation of activity by 27% ± 6%) and 1.15 ± 0.06 for ^90^Y-loaded resin microspheres (i.e. underestimation of activity by − 13% ± 4%) when vials were measured by PET/CT imaging [10]. Those results have recently been remarkably confirmed by Monte-Carlo simulations for, respectively, ^90^Y-loaded glass microspheres (i.e. overestimation of the activity by 25% ± 4%) and ^90^Y-loaded resin microspheres (i.e. underestimation of activity by − 15% ± 2%) when using vial measurement [11].

In the present in-patient study, we found an average A_PET_/A_calibrator_ of 0.90 ± 0.06 for ^90^Y-loaded glass microspheres (i.e. overestimation of activity by 12% ± 7%) and 1.30 ± 0.11 for ^90^Y-loaded resin microspheres (i.e. underestimation of activity by − 22% ± 7%) when considering patients imaged by ^90^Y-PET/CT after SIRT (when using ABS scatter scaling, which is considered more reliable for extremely low counts, as in ^90^Y imaging). In patients imaged by PET/MR, the mean A_PET_/A_calibrator_ was 0.76 ± 0.07 for ^90^Y-loaded glass microspheres (i.e. overestimation of activity by 33% ± 14%). These results of the ratio A_PET_/A_calibrator_ are in moderate agreement with the PET results of the vial experiment, although the trend was similar between ^90^Y-loaded glass and resin microspheres when considering the average relative activity difference between resin and glass microspheres, i.e. 44% in the current study compared to 46% for the vial experiment [10] and 46.5% for the vial Monte Carlo study [11]. Any discrepancies between the vial and patient measurements could be mainly attributed to the different action of the scatter scaling between the vial and patient conditions, the first characterized by a highly concentrated activity in a small volume with low scatter medium, the latter by a relatively low activity concentration distributed in a larger diffusive medium (the perfused liver). Furthermore, we found a significant difference between the TFBS and ABS scatter scaling approaches for both PET/CT and PET/MR using ^90^Y-loaded glass microspheres (Supplemental Figure S3). The TFBS approach systematically overestimated the scatter fraction compared to the ABS approach (Supplemental Figure S3). More importantly, the variability (and outliers) of the scatter fraction computed using TFBS was more pronounced when patients were categorized according to their BMI (Supplemental Figures S4 and S5) although a low variability is expected within each BMI category. This effect tends to be more pronounced for the PET/MR system, likely due to the lack of TOF capability and the smallest radial FOV (Supplemental Figure S5). These results suggest that TFBS is unreliable in this extreme condition of low count for evaluating the scaling factor and should be systematically avoided when using both PET/CT and PET/MR for ^90^Y imaging.

A significant difference was also found between PET/MR and PET/CT although the patients were not the same (except for one). The mean A_PET_/A_calibrator_ for PET/CT was approximately 18% higher than for PET/MR. This trend is consistent with the results of a previous pilot study exploring 32 patients who were imaged twice in a row (once with PET/CT and once with PET/MR) [16]. The authors reported a significant underestimation of mean AD to the liver when comparing PET/MR with PET/CT (^90^Y-loaded glass and resin microspheres mixed), ranging from 2.4% to 18.5% (although the scatter scaling approach was not mentioned for either PET/CT or PET/MR). The difference between the two systems is more likely due to the lack of TOF capability and the smallest radial FOV for PET/MR compared to PET/CT. In this context, it may be more difficult to accurately scale the scatter component in a very low statistics regime.

It is worth noting that the reported relative activity differences between ^90^Y-loaded glass and resin microspheres will not affect current patient management in terms of safety and treatment efficacy for the two types of microspheres. However, it may be an important piece of information in the context of comparing the two devices in terms of tumour control probability [17]. Harmonizing the metrology chain of ^90^Y devices with a common standard reference (e.g. using quantitative PET/CT imaging or dose calibrator correction factors derived from Monte-Carlo simulations) became key when considering the transition of patients from one treatment option to another, i.e. from resin to glass or vice versa, or addition of External Beam Radiation Therapy. Finally, to comply with the ICRU level 1 recommendation (minimum standards for prescribing and reporting [18]), the therapeutic administration of activity must be known to within 10% of the intended value [19].

Based on the results of the PET vials experiments [11] and considering the present in-liver PET data, we can postulate that the previously reported AD levels for toxicity and response in resin and glass microsphere SIRT are closer to each other than previously reported. Such ADs have been derived from predictive dosimetry based on ^99m^Tc-MAA SPECT/CT imaging, where the intended therapeutic activity is distributed proportionally to the signal intensity in the image voxels. For example, when treating HCC with SIRT in a lobar approach (following a multi-compartment approach), the AD thresholds for normal liver associated with safety are 40 Gy and 120 Gy, whereas the tumor AD associated with improved response are 120 Gy and 205 Gy, for resin and glass microsphere approaches respectively [5]. Considering the current results, these AD levels would translate into 52 Gy (40 × 1.30) and 108 Gy (120 × 0.90) for safety and 156 Gy and 185 Gy for efficacy for resin and glass microspheres respectively. Further clinical results are needed to verify this statement.

This study also advocates for an improved scatter correction approach, given the relatively high standard deviation of A_PET_/A_calibrator_ reported in this work. Moreover, dosimetry for SIRT is prevalent and increasingly relied upon. Personalized dosimetry has led to improved local control, progression free survival, and overall survival. With such gains, the number of patients receiving additional therapy will increase. Improved harmonization and precision of the reconstructed activity distribution and AD calculation will aid in eventual pooling and assessing of long-term toxicity as well as in studies with combination therapies. For example, the Michigan group is using the ^90^Y-PET/CT derived AD maps to deliver Stereotactic Body Radiation Therapy (SBRT) boosts to lesions underdosed by SIRT (NCT04518748).

In this study, a ^90^Y phantom reference experiment to verify the alignment of ^90^Y-PET quantitative assessment was not performed across participating centres logistical reasons. Nevertheless, PET/CT-1, PET/CT-3 and PET/CT-4 PET scanners are EARL accredited (Standard 2), while PET/CT-2 is ACR® Qualification Utility for Imaging Core lab (QUIC) certified. In fact, their accuracy in measuring ^18^F sources is harmonized. The quantitative scaling from ^18^F calibration to ^90^Y quantitative assessment is uniform across the PET devices used, so we do not expect any relevant inter-device variation in ^90^Y quantitative performance.

A notable limitation of this study arises from its reliance on measurements obtained from dose calibrators, which are known to be susceptible to uncertainties [20]. Although efforts have been made to minimize experimental errors and to standardize measurement procedures, the inherent limitations of the calibrators themselves introduce a degree of uncertainty into the comparative analysis of ^90^Y measurements. Consequently, the conclusions drawn from this study are subject to the limitations associated with the accuracy and reliability of the dose calibrators used in the experiments. Our experience of measuring the same vials at different activity levels using the three methods (namely dose calibrator, dose-rate and ^90^Y PET) shows similar levels of accuracy. These data are not currently published. Furthermore, ^90^Y-PET offers advantages over the dose calibrator and dose-rate methods, such as better spatial uniformity, reduced sensitivity to the specific source geometry and composition. We therefore consider quantitative ^90^Y-PET to be a technique for measuring residual ^90^Y activity that is as accurate as, or potentially superior to, exposure rate or dose calibrator measurements.

## Conclusions

This study confirmed in SIRT patient data that the activity of ^90^Y-loaded microspheres consistently differed from that declared (and measured locally) by the manufacturer. A large relative difference of 44% was found between the mean ratio A_PET_/A_calibrator_ of glass ^90^Y-loaded microspheres (mean ratio of 0.90) and ^90^Y-loaded resin microspheres (mean ratio of 1.30), like that previously reported from PET vial measurements (46%) and confirmed by Monte Carlo simulations. Significant difference between PET/CT and PET/MR was observed, indirectly confirming the benefit of TOF for an accurate quantification and the use of a larger radial FOV for a reliable scatter estimation. The scatter correction approach significantly impacted the quantification: the tail-fitting background scaling should be avoided.

## Supplementary Information

Additional file 1.

## Data Availability

The datasets generated during and/or analysed during the current study are available from the corresponding author on reasonable request.
