# Supplementary material for: Discordance between 90Y-PET/CT(MR)-estimated activity and dose calibrator measured administered activity: an international study in SIRT patients treated with resin and glass microspheres
Source: EJNMMI Phys. 2025 Feb 5;12:12. doi: 10.1186/s40658-025-00725-8 (PMC11799454; doi:10.1186/s40658-025-00725-8)
Supplement: Supplementary file 1 — Additional file 1. [file 40658_2025_725_MOESM1_ESM.docx]

**Supplemental Table S1.** Acquisition and reconstruction parameters for each system.

| **PET ID** | **Device** | **Centre** | **Acquisition parameters** | **Reconstruction parameters** |
| --- | --- | --- | --- | --- |
| PET/CT-1 | Siemens Biograph Vison 600 | CHUV Lausanne | List-mode 15 minutes (step and shoot) | OP-OSEM3D TOF+PSF  2 iterations × 5 subsets + Gaussian post-filtering (FWHM=4mm)  Voxel size=3.3x3.3x1.65 mm^3^ |
| PET/CT-2 | Siemens Biograph mCT 40 | University of Michigan Hospital | List-mode 20 minutes (step and shoot) | OP-OSEM3D TOF+PSF  2 iterations × 21 subsets + Gaussian post-filtering (FWHM=5mm)  Voxel size=4×4×2 mm^3^ |
| PET/CT-3 | Siemens Biograph Vison 600 | Luzerner Kantonsspital | List-mode 15 minutes (step and shoot) | OP-OSEM3D TOF+PSF  2 iterations × 5 subsets + Gaussian post-filtering (FWHM=4mm)  Voxel size=3.3x3.3x1.65 mm^3^ |
| PET/CT-4 | Siemens Biograph Vison 600 | University Hospital of Nantes | List-mode 30 minutes (step and shoot) | OP-OSEM3D TOF+PSF  2 iterations × 5 subsets + Gaussian post-filtering (FWHM=4mm)  Voxel size=3.3×3.3×1.65 mm^3^ |
| PET/MR-1 | Siemens Biograph mMR | University Hospital of Nantes | List-mode 30 minutes (step and shoot) | OP-OSEM3D PSF  3 iterations × 21 subsets + Gaussian post-filtering (FWHM=4mm)  Voxel size=4.2×4.2×2 mm^3^ |

**Supplemental Table S2.** Methods used for LSF and residual fraction for each center

| **Institution** | **LSF measurement** | **Residual fraction measurement** |
| --- | --- | --- |
| CHUV | Planar imaging | PET/CT imaging |
| University of Michigan | Planar imaging | Survey meter |
| Luzerner Kantonsspital | SPECT/CT imaging | PET/CT imaging |
| University Hospital of Nantes | SPECT/CT imaging | PET/CT imaging |


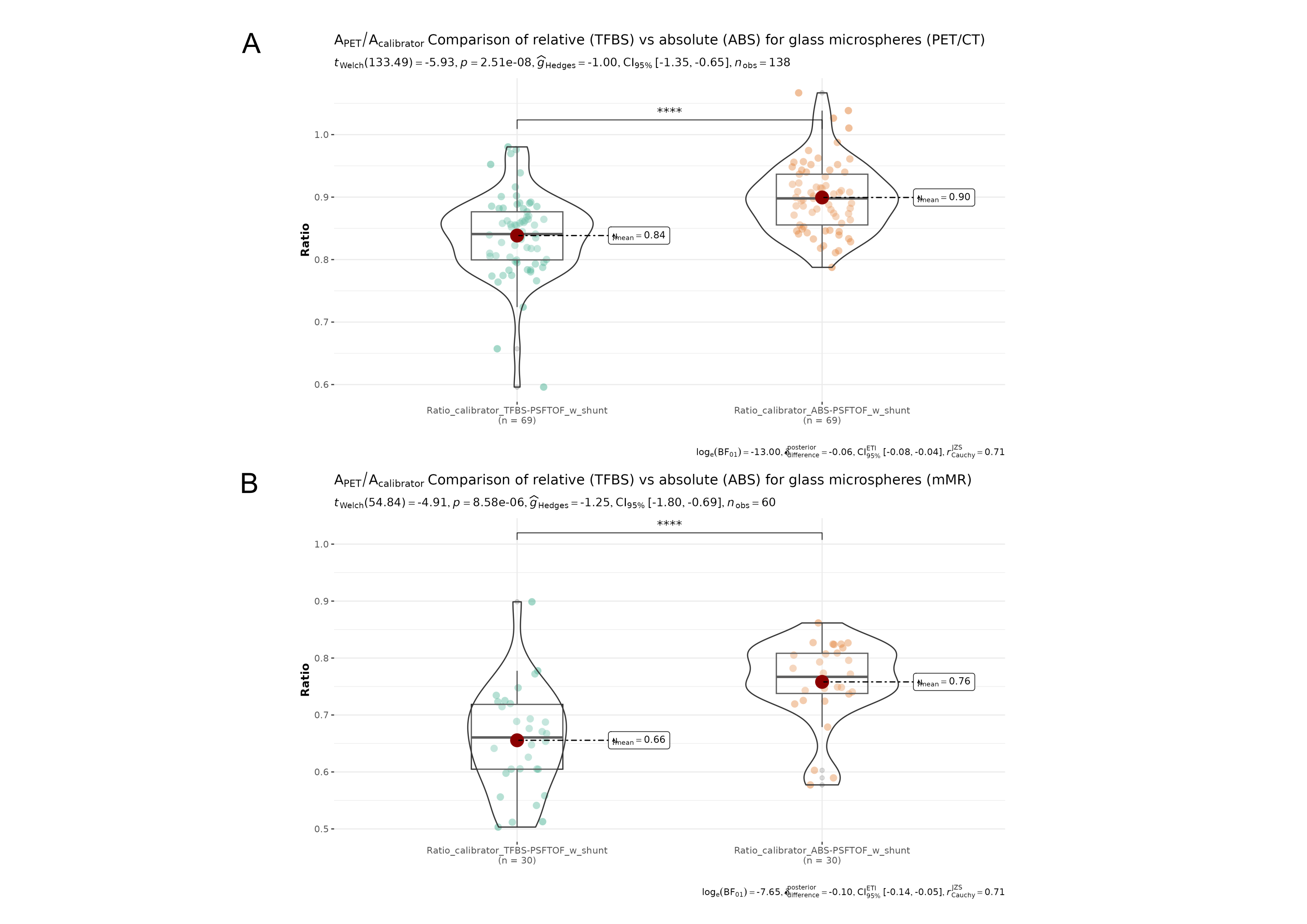


**Supplemental Figure S1.** Comparison of A_PET_/A_calibrator_ between the TFBS and ABS scatter scaling approach for PET/CT (A) and PET/MR (B) using ^90^Y-loaded glass microspheres.

**Supplemental Figure S2.** Bland-Altman plots for comparison of scatter fraction using TFBS and ABS for PET/CT (A) and PET/MR (B). Green area is the upper limit of agreement (+1.96xSD) with the 95% confidence interval (CI), where SD is the standard deviation. Red area is the lower limit (-1.96xSD) of agreement (+1.96xSD) with the 95% CI. Purple area is the mean difference with the 95% CI.


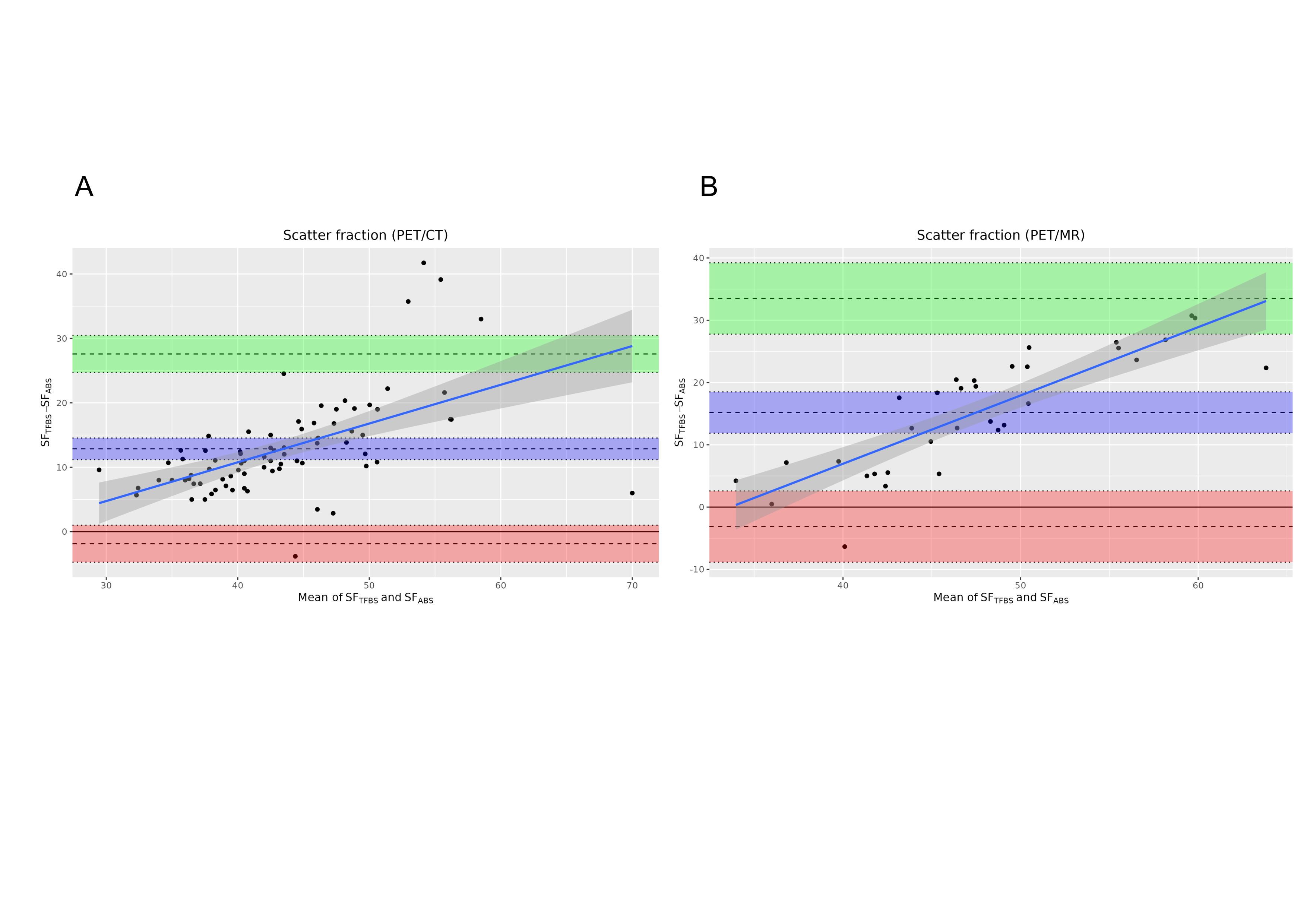


**Supplemental Figure S3.** Comparison of scatter fraction the TFBS and ABS scatter scaling approach for PET/CT (A) and PET/MR (B) using ^90^Y-loaded glass microspheres.


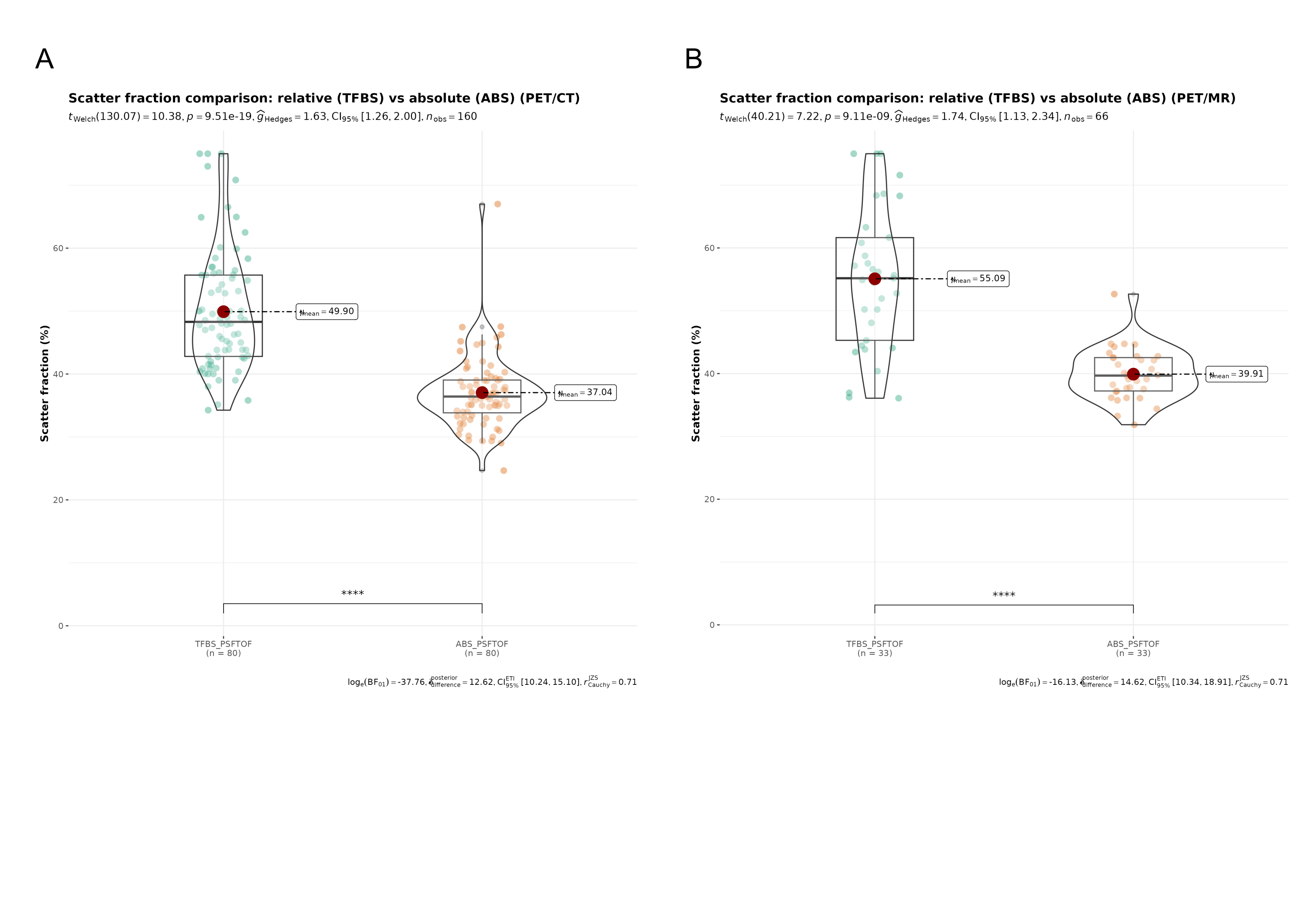


**Supplemental Figure S4.** Scatter fraction computed using tail-fitting background scaling (TFBS) and absolute scaling (ABS) as a function of the BMI (Normal: BMI ∊ [18.5-25] kg.m^-2^, Overweight: BMI ∊ ]25-30] kg.m^-2^, Obesity: BMI >30 kg.m^-2^) for PET/CT.


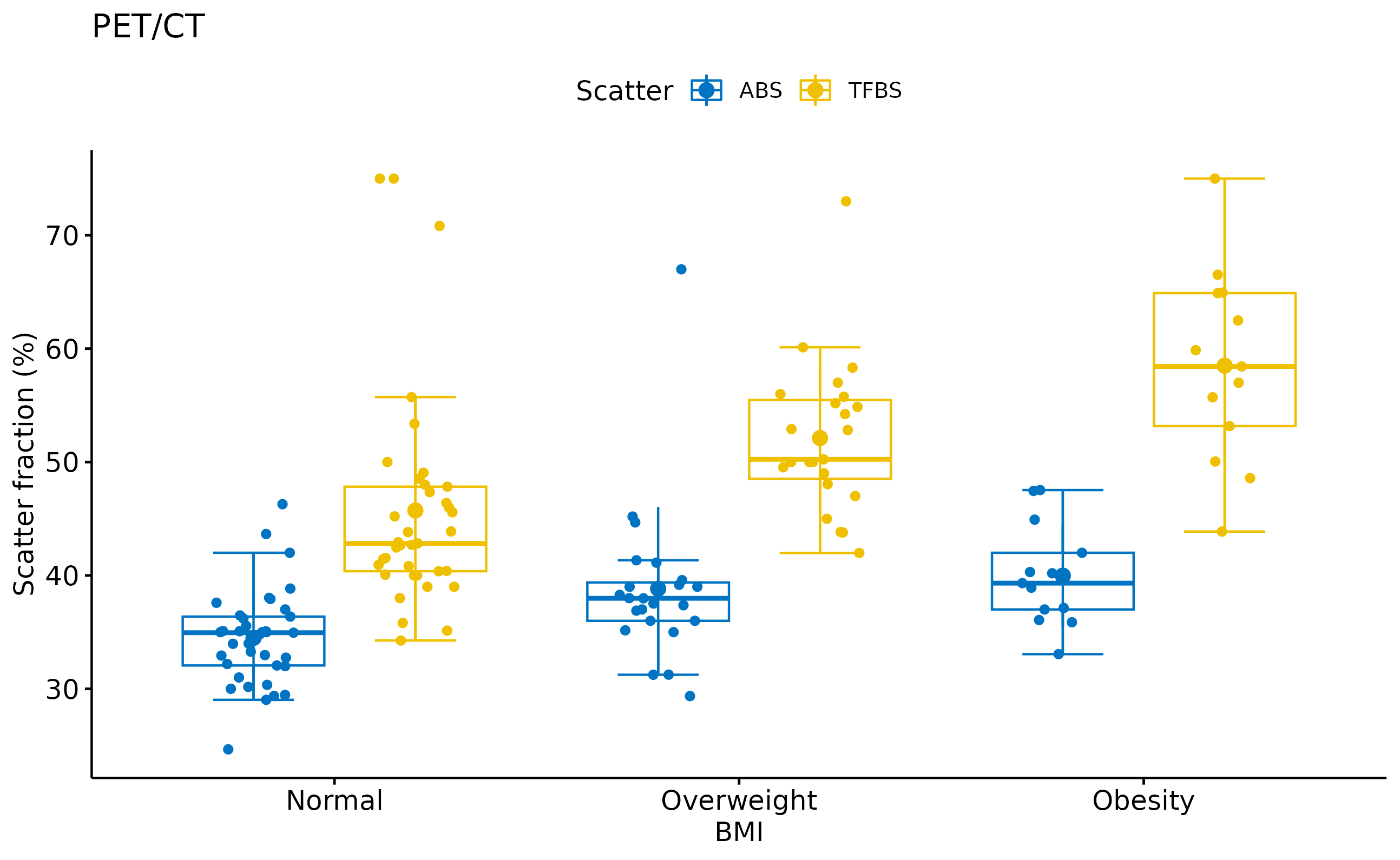


**Supplemental Figure S5.** Scatter fraction computed using tail-fitting background scaling (TFBS) and absolute scaling (ABS) as a function of the BMI (Normal: BMI ∊ [18.5-25] kg.m^-2^, Overweight: BMI ∊ ]25-30] kg.m^-2^, Obesity: BMI >30 kg.m^-2^) for PET/MR.


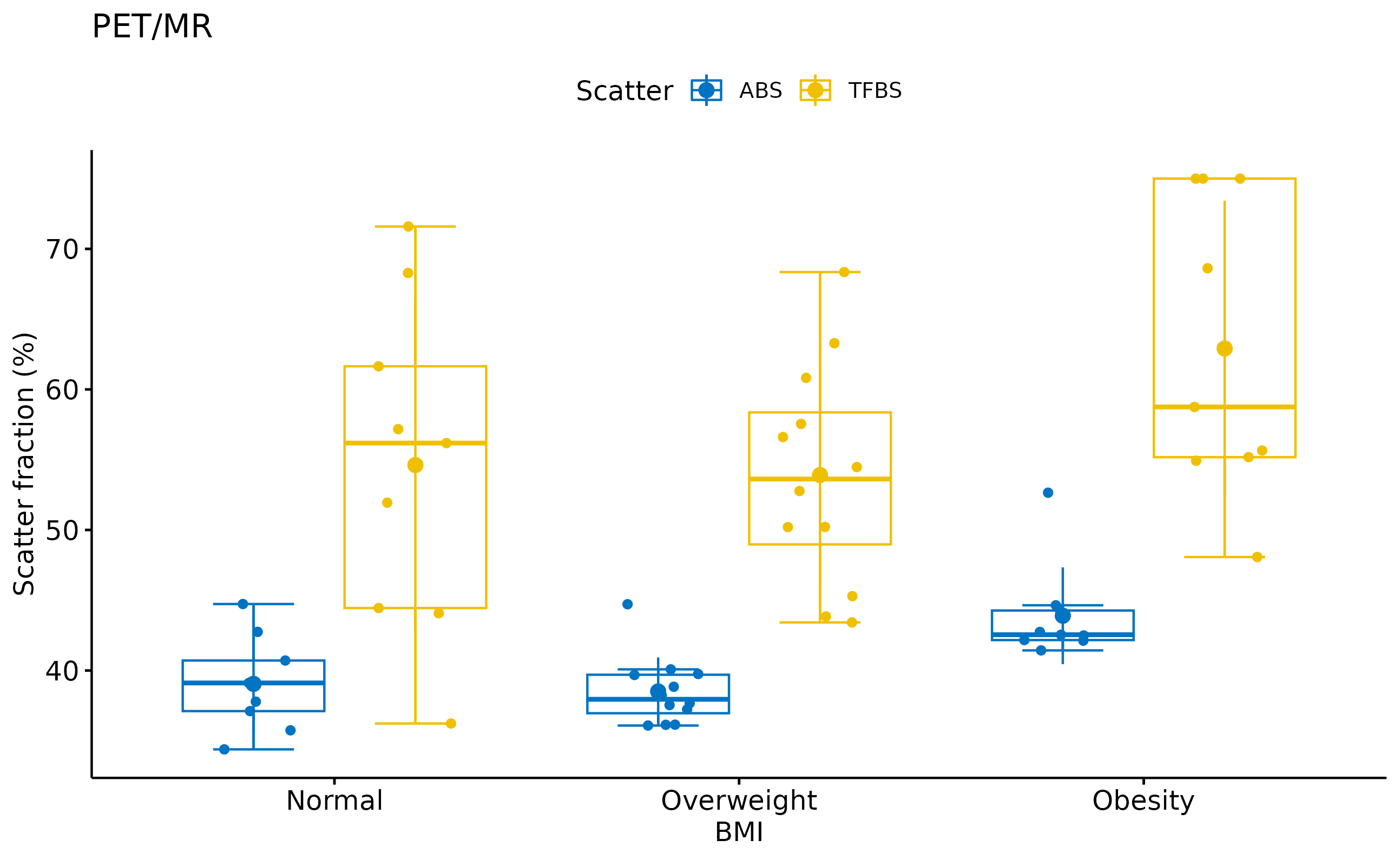

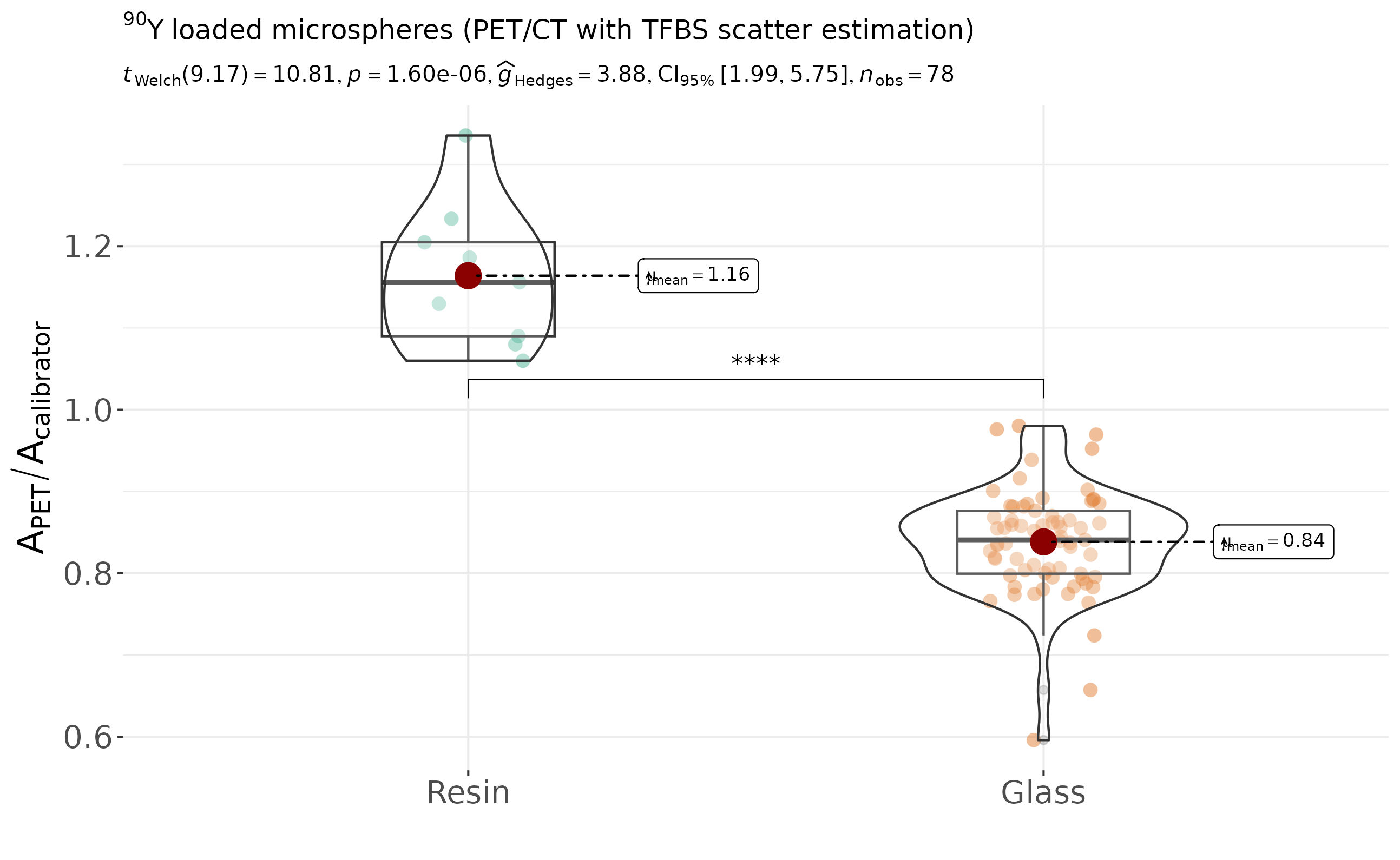


**Supplemental Figure S6.** Comparison of A_PET_/A_calibrator_ between patients treated with ^90^Y-loaded glass and resin microspheres using a PET/CT (relative rescaling for scatter estimation).
